# Supplementary material for: Synthesis, Molecular Docking Study, and Cytotoxicity Evaluation of Some Novel 1,3,4-Thiadiazole as Well as 1,3-Thiazole Derivatives Bearing a Pyridine Moiety
Source: Molecules. 2022 Sep 27;27(19):6368. doi: 10.3390/molecules27196368 (PMC9572991; doi:10.3390/molecules27196368)
Supplement: Supplementary file 1 [file molecules-27-06368-s001.zip › molecules-1920307-supplementary.pdf]

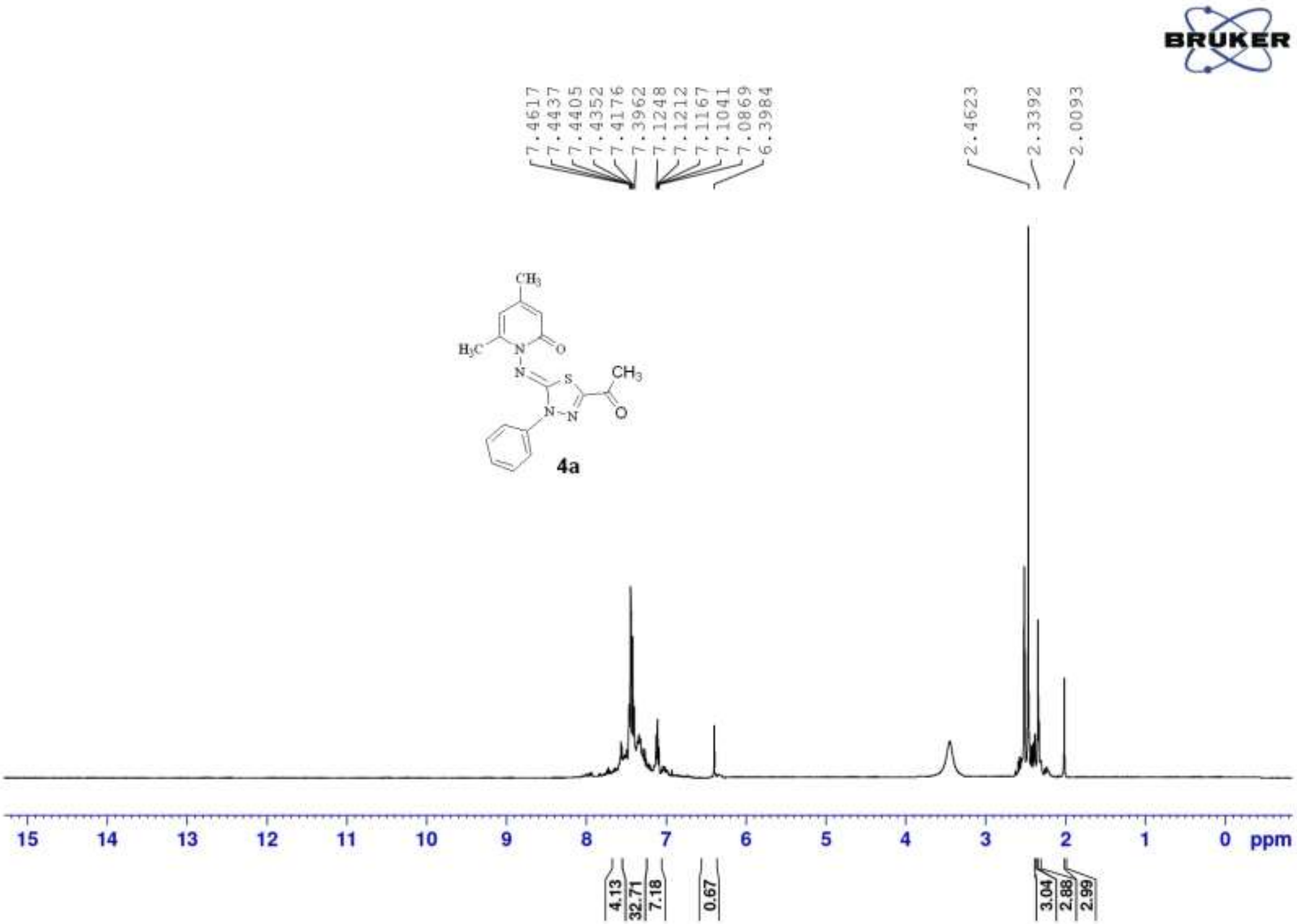

^{1}H-NMR of compound **4a**

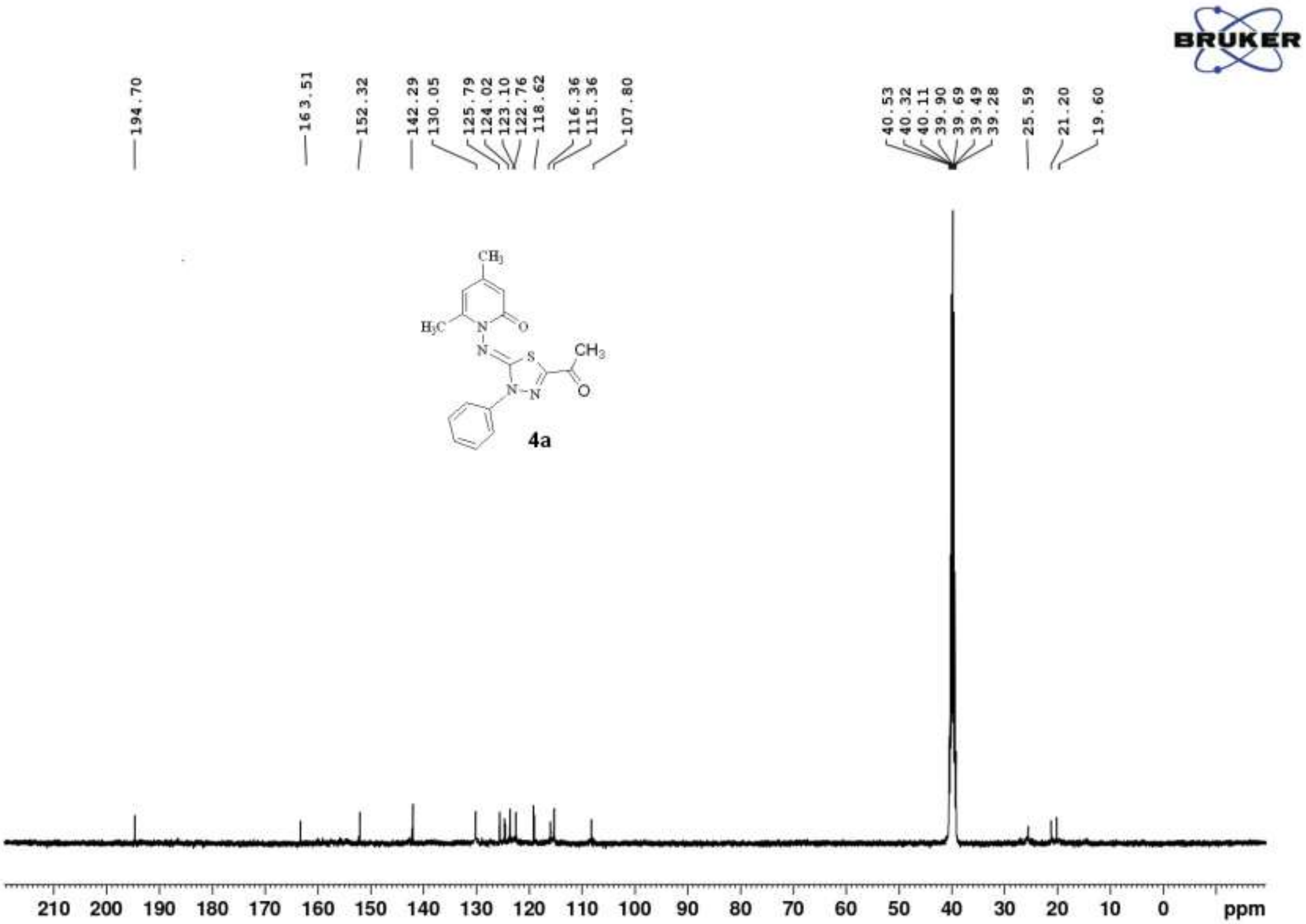

^{13}C-NMR of compound **4a**

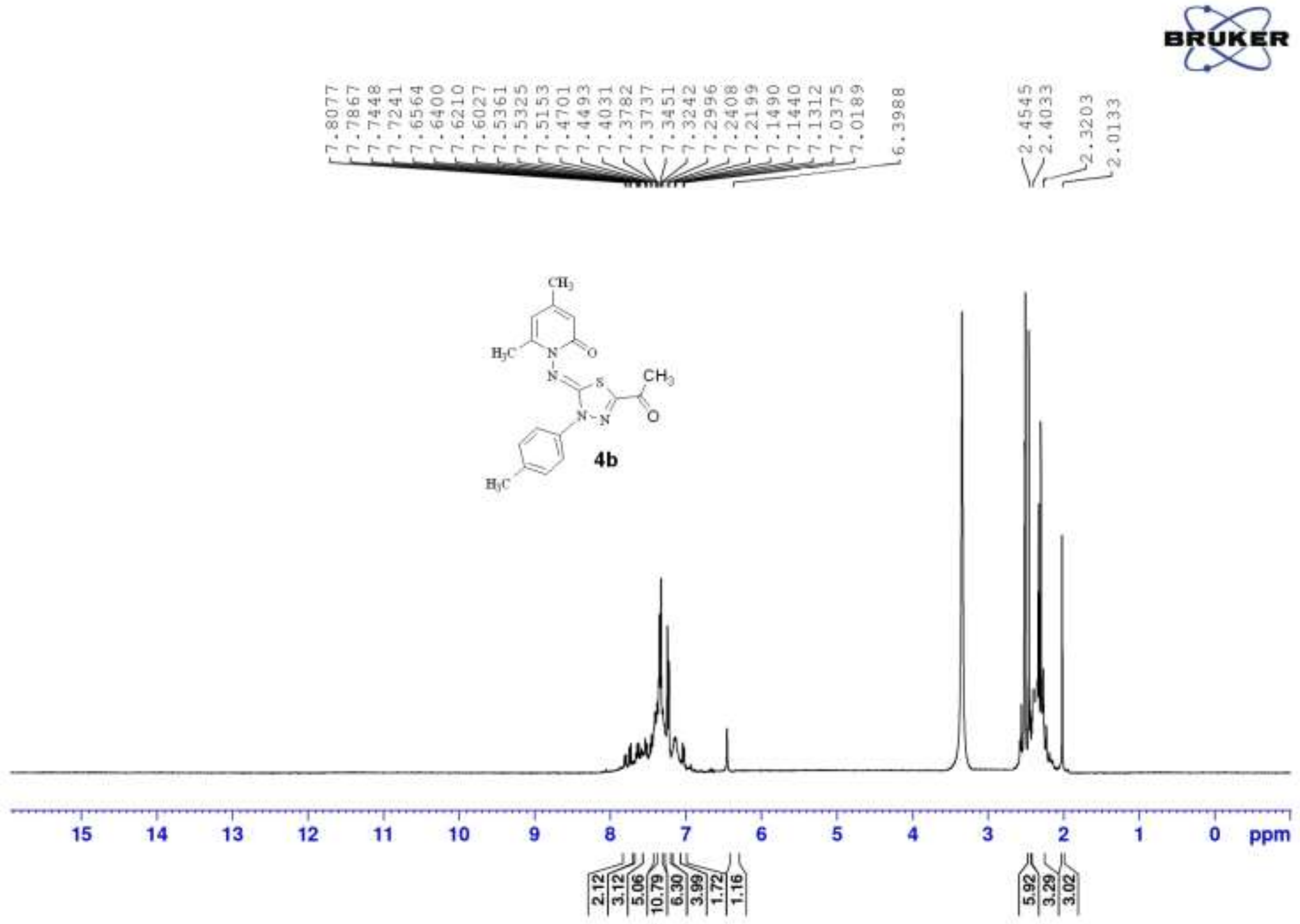

^{1}H-NMR of compound **4b**

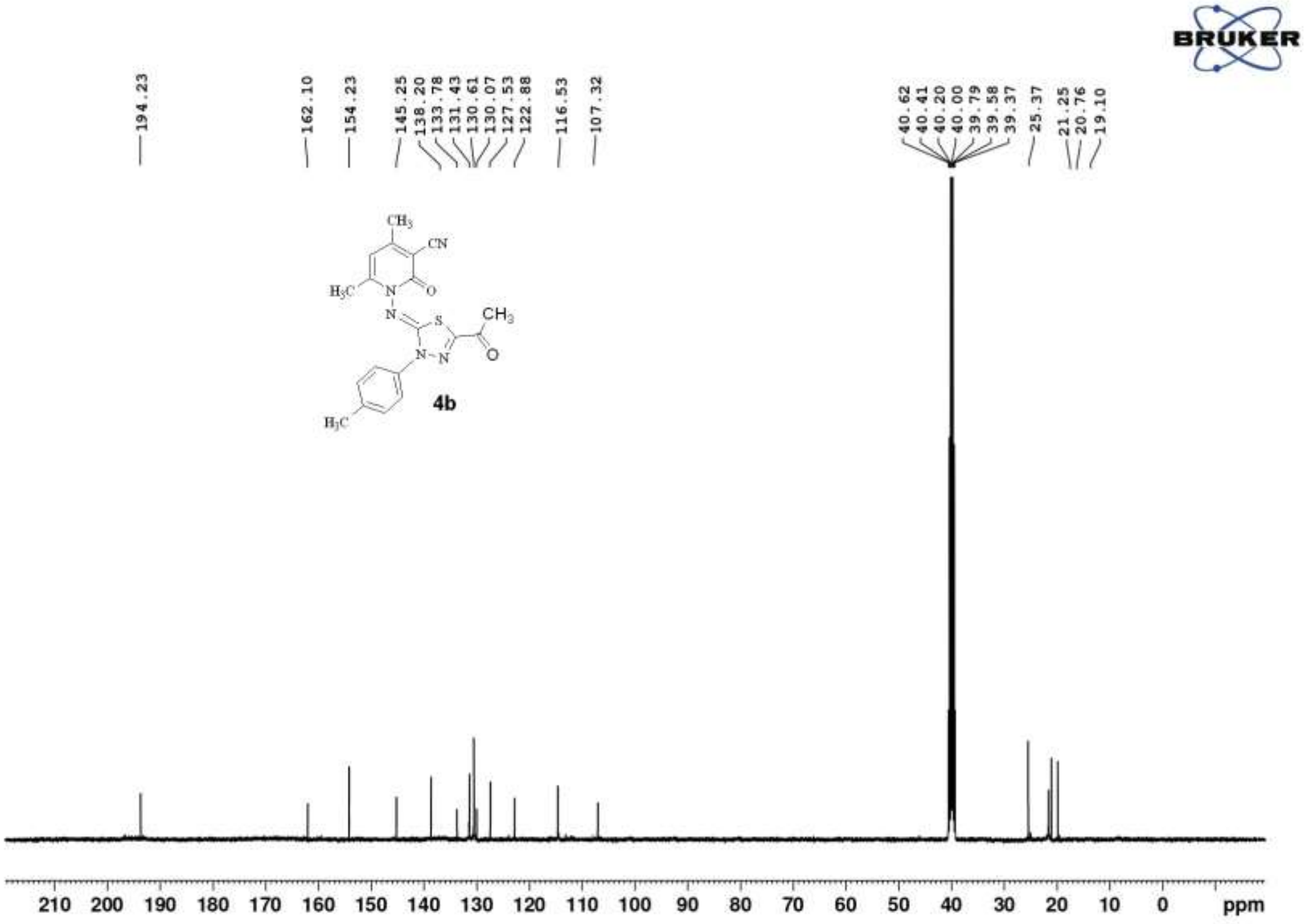

^{13}C-NMR of compound **4b**

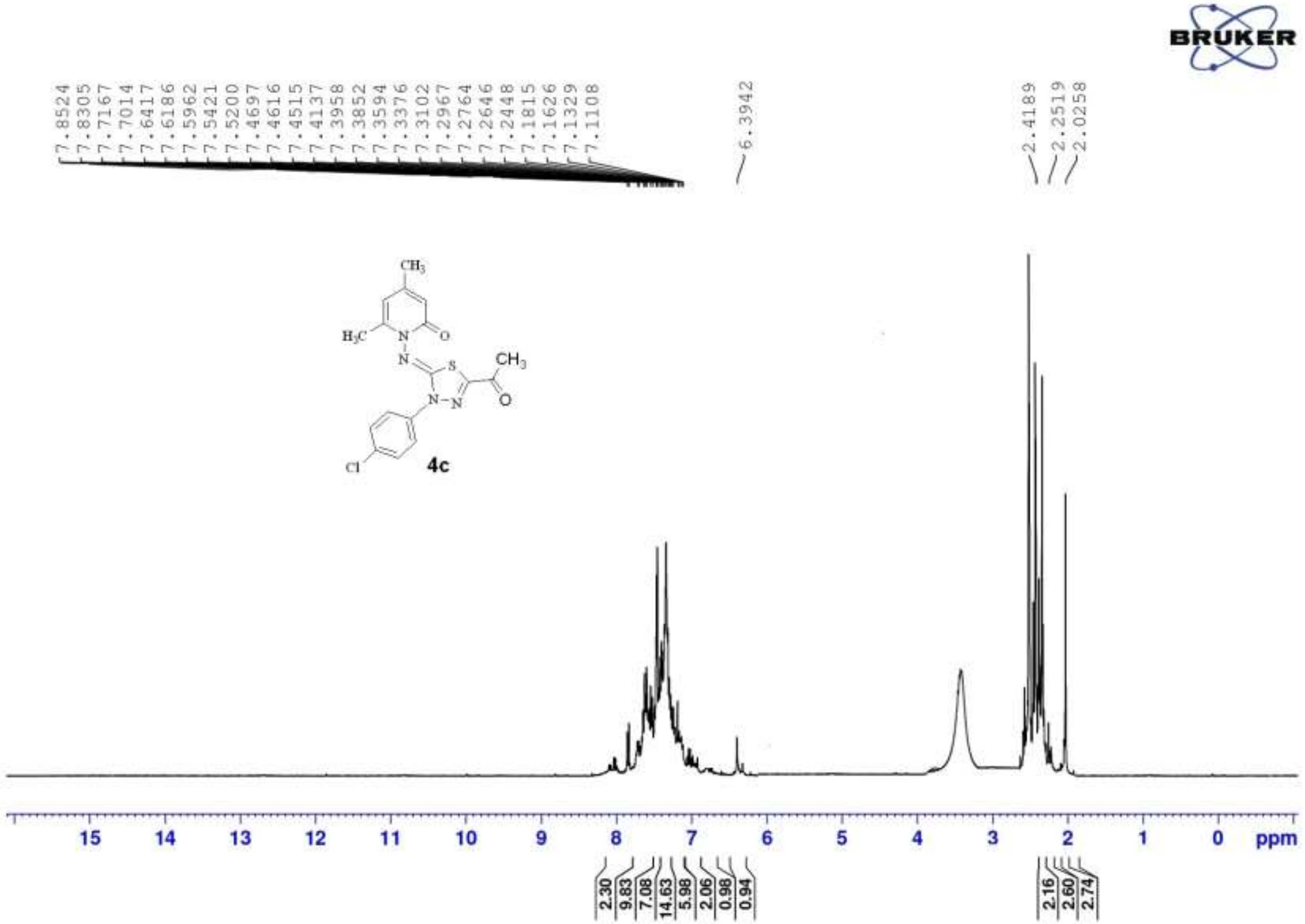

^{1}H-NMR of compound **4c**

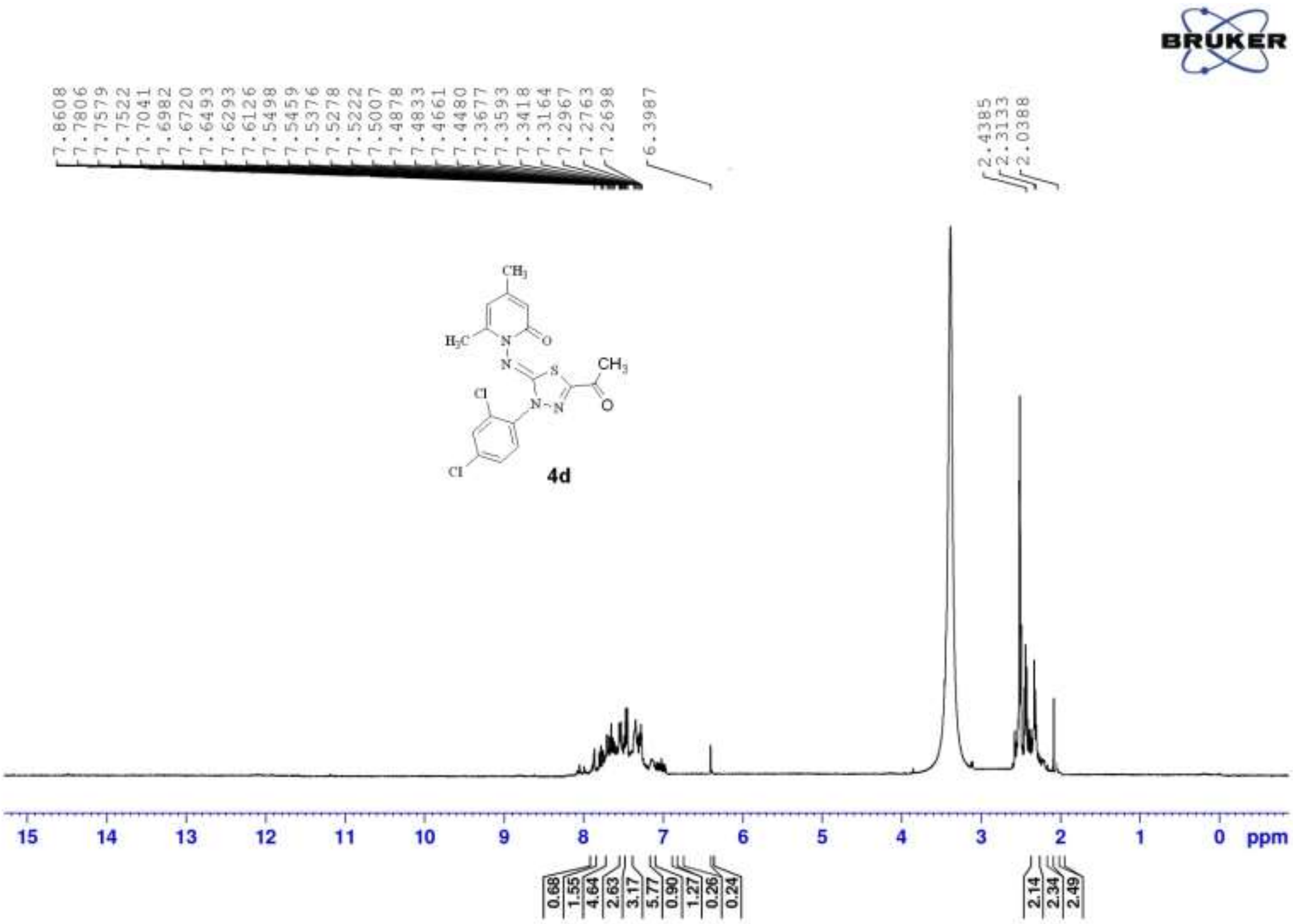

^{1}H-NMR of compound **4d**

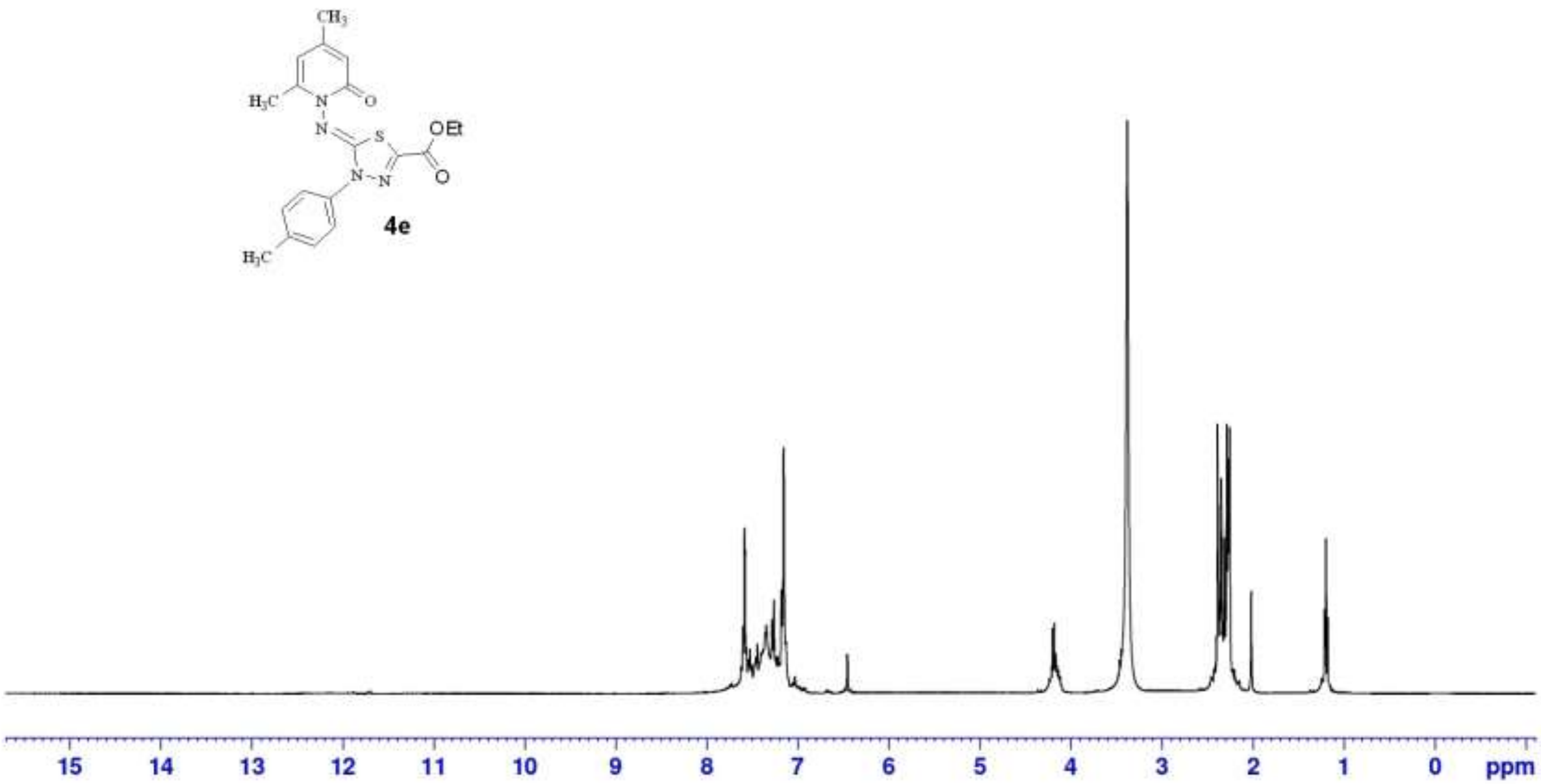

^{1}H-NMR of compound **4e**

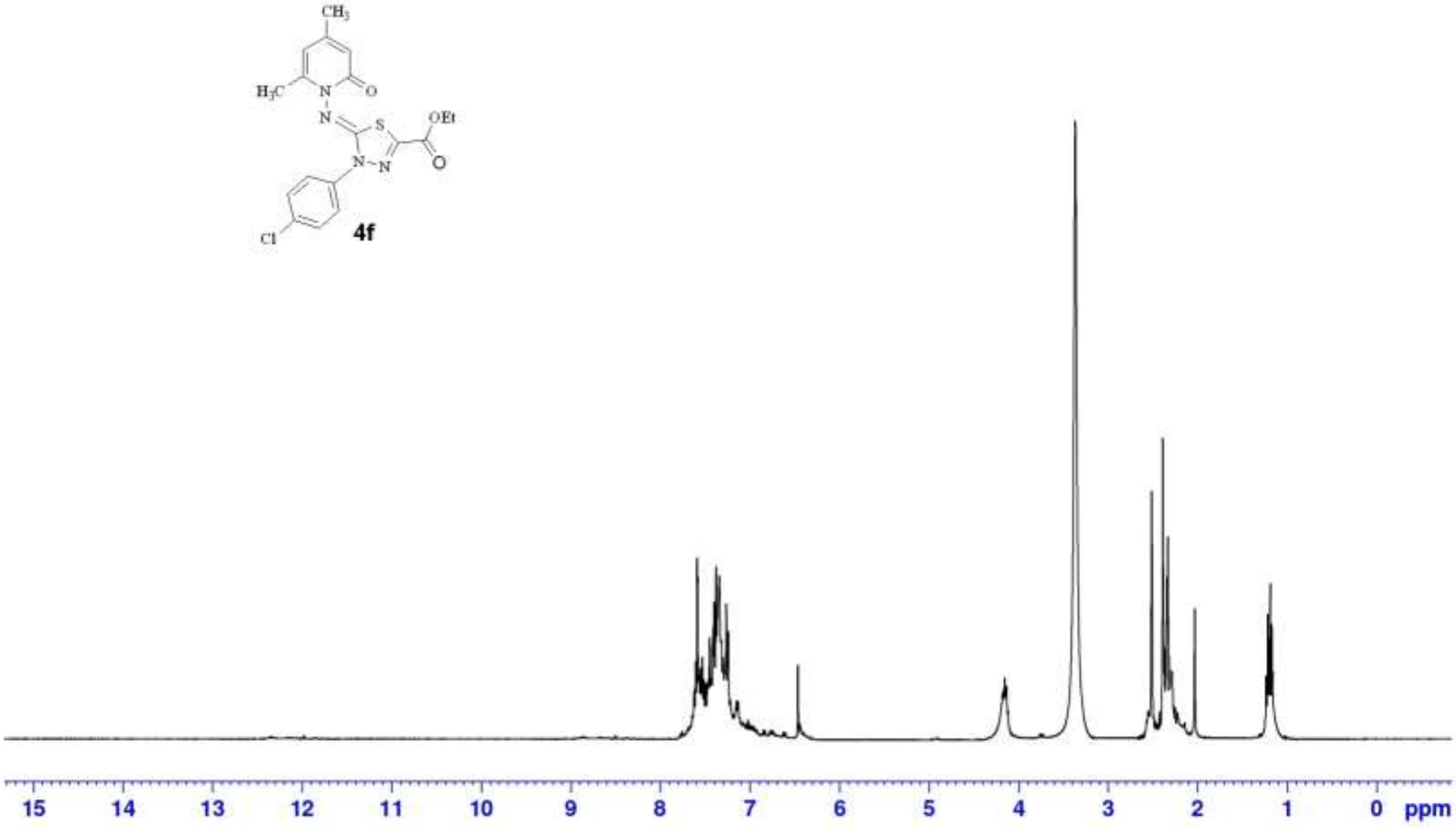

^{1}H-NMR of compound **4f**

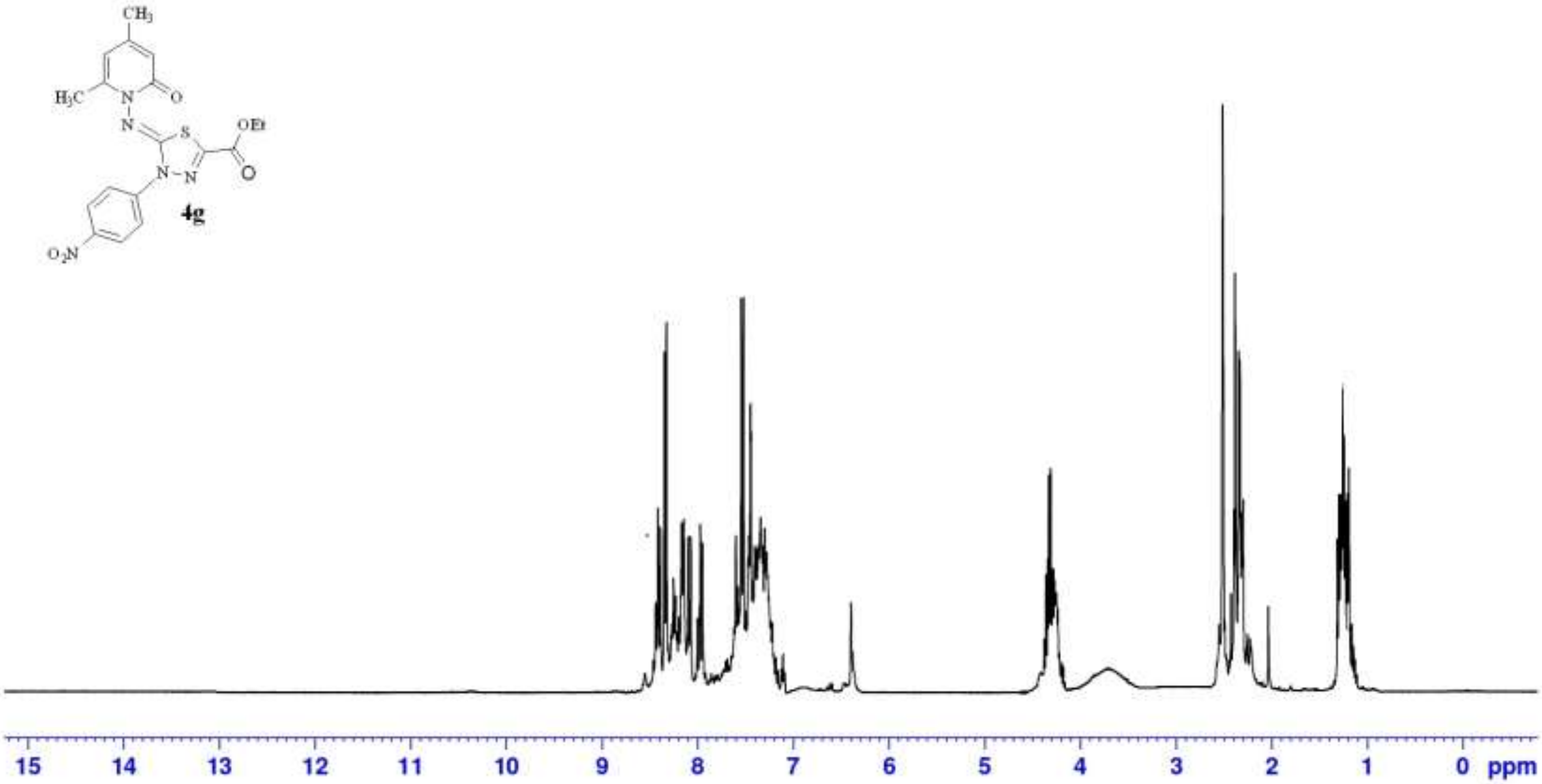

^{1}H-NMR of compound **4g**

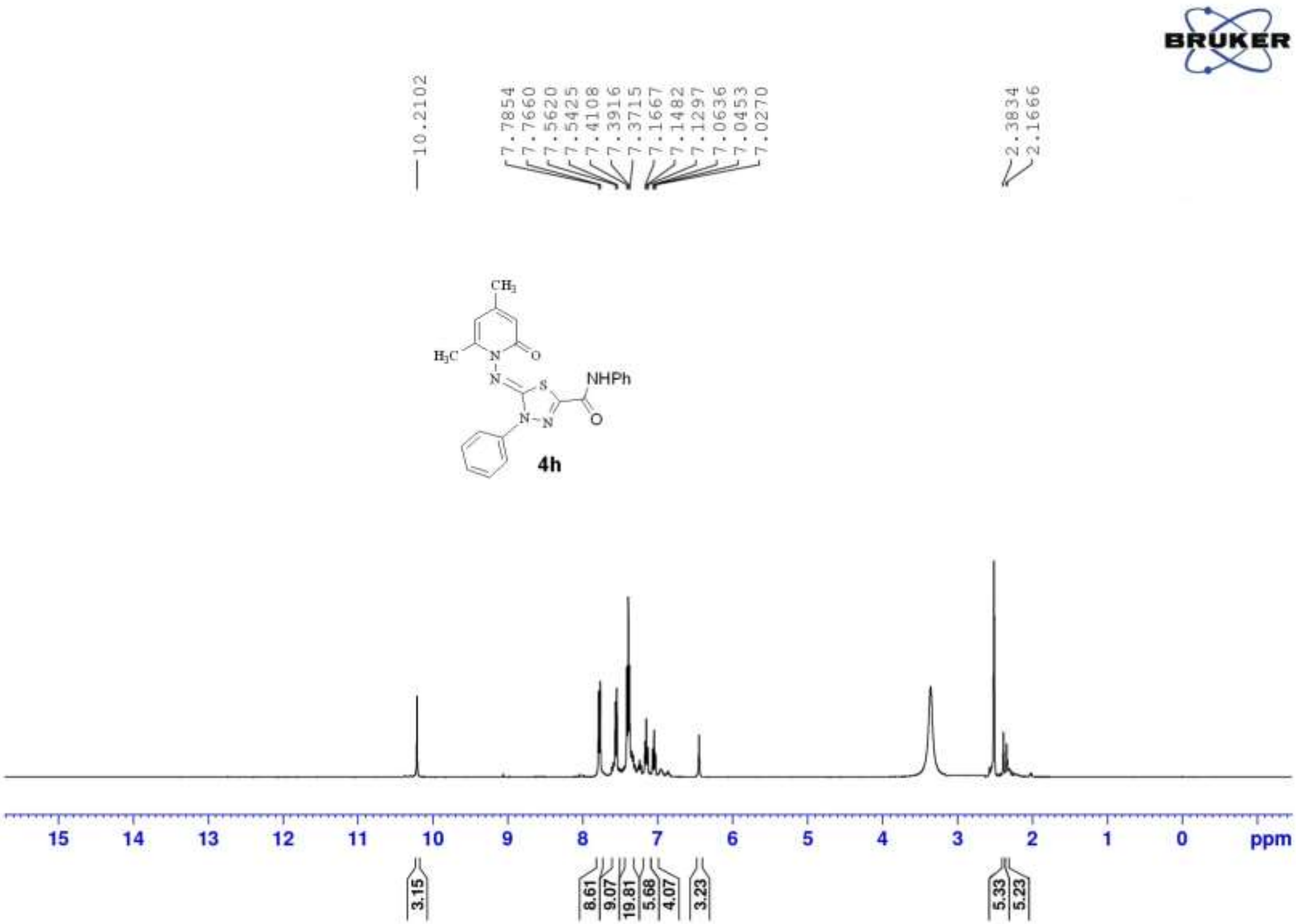

^{1}H-NMR of compound **4h**

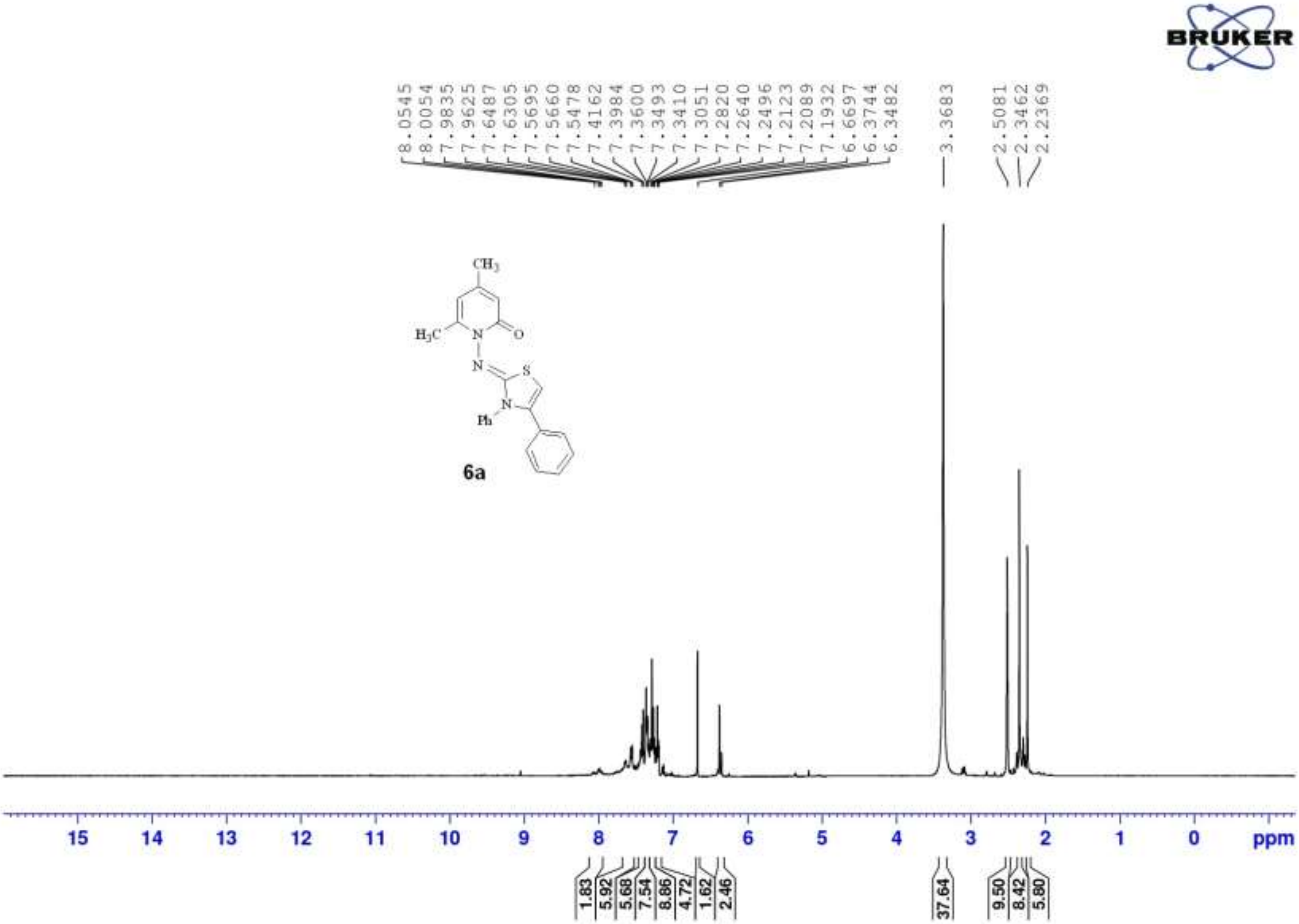

^{1}H-NMR of compound **6a**

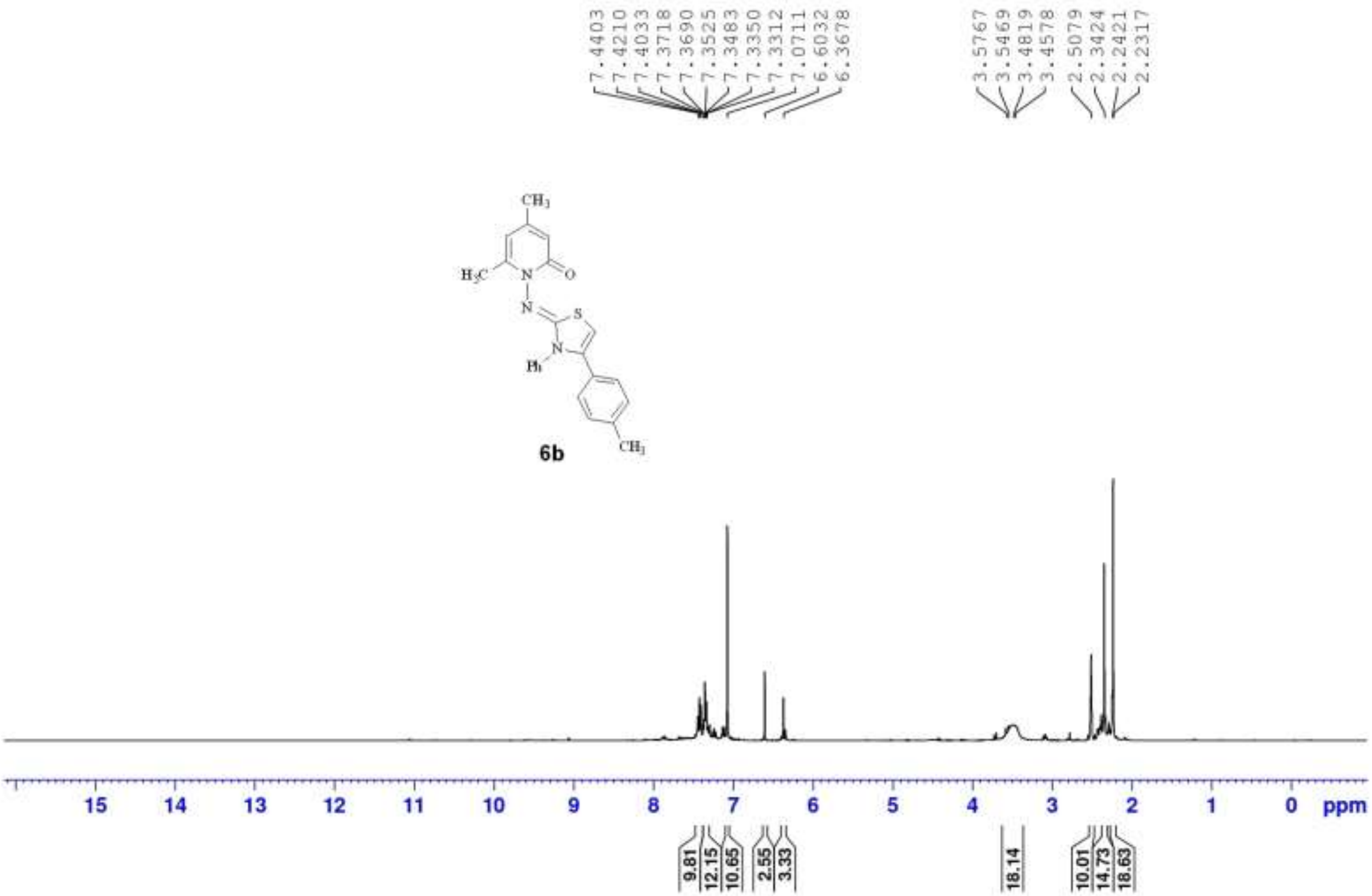

^{1}H-NMR of compound **6b**

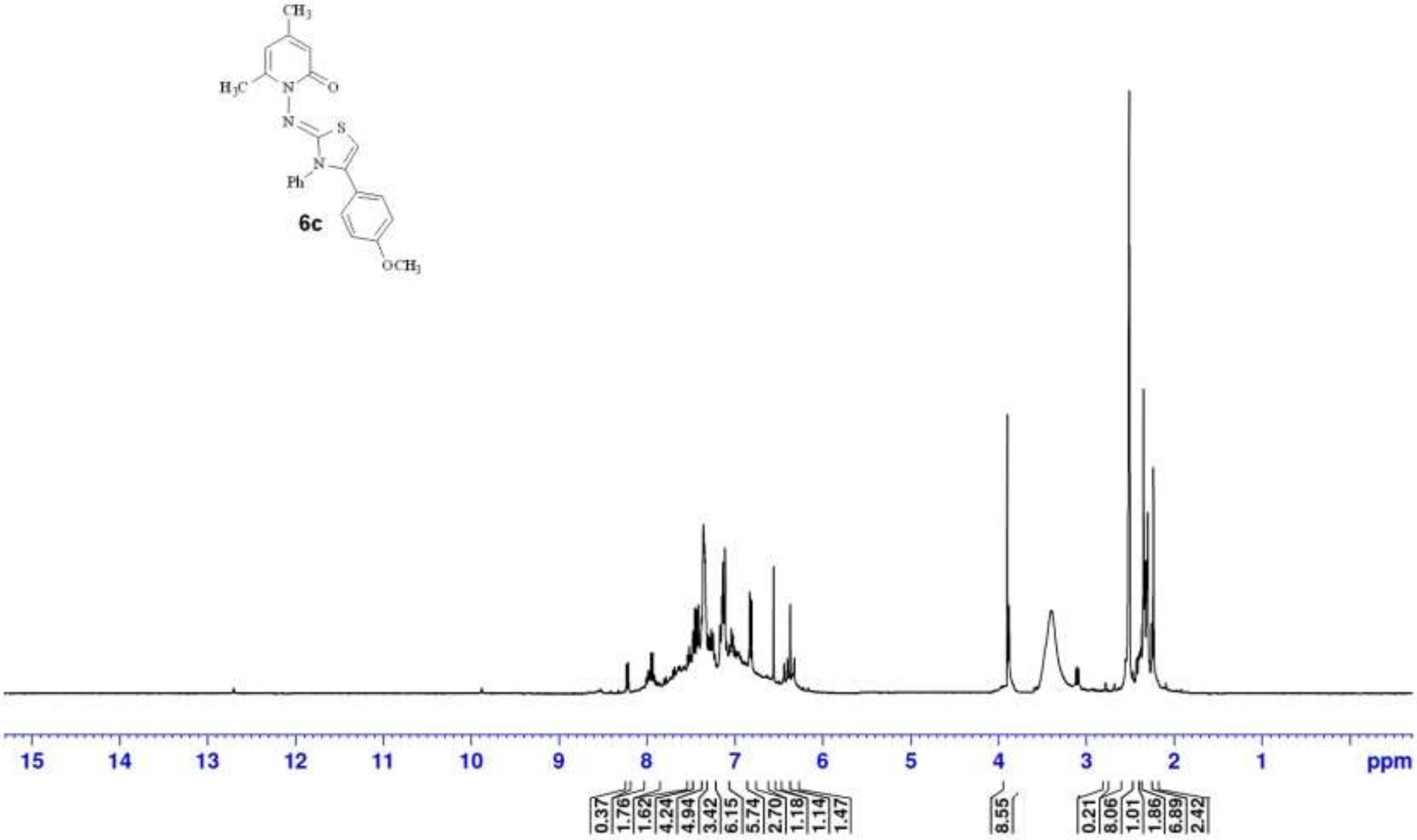

^{1}H-NMR of compound **6c**

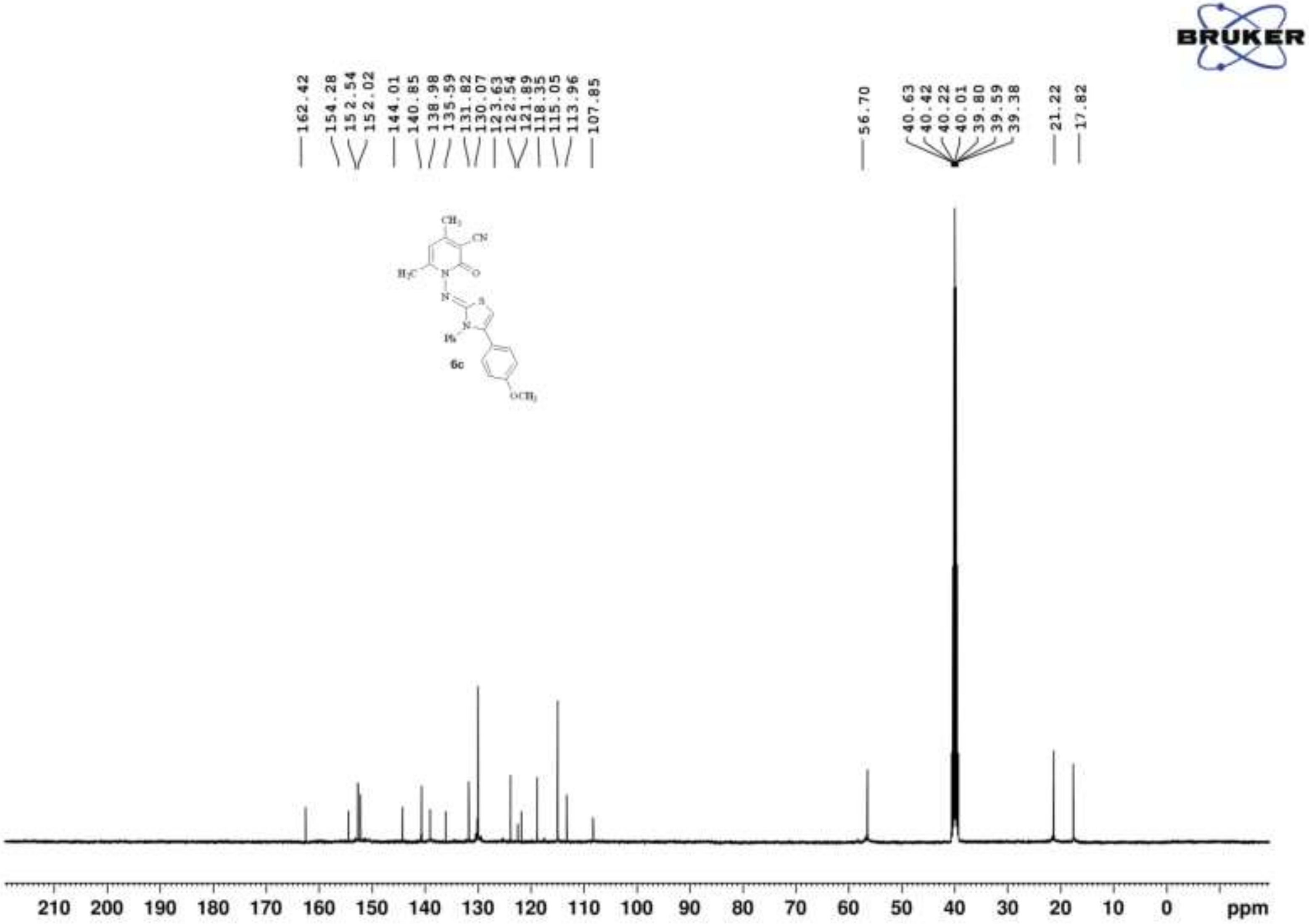

^{13}C-NMR of compound **6c**

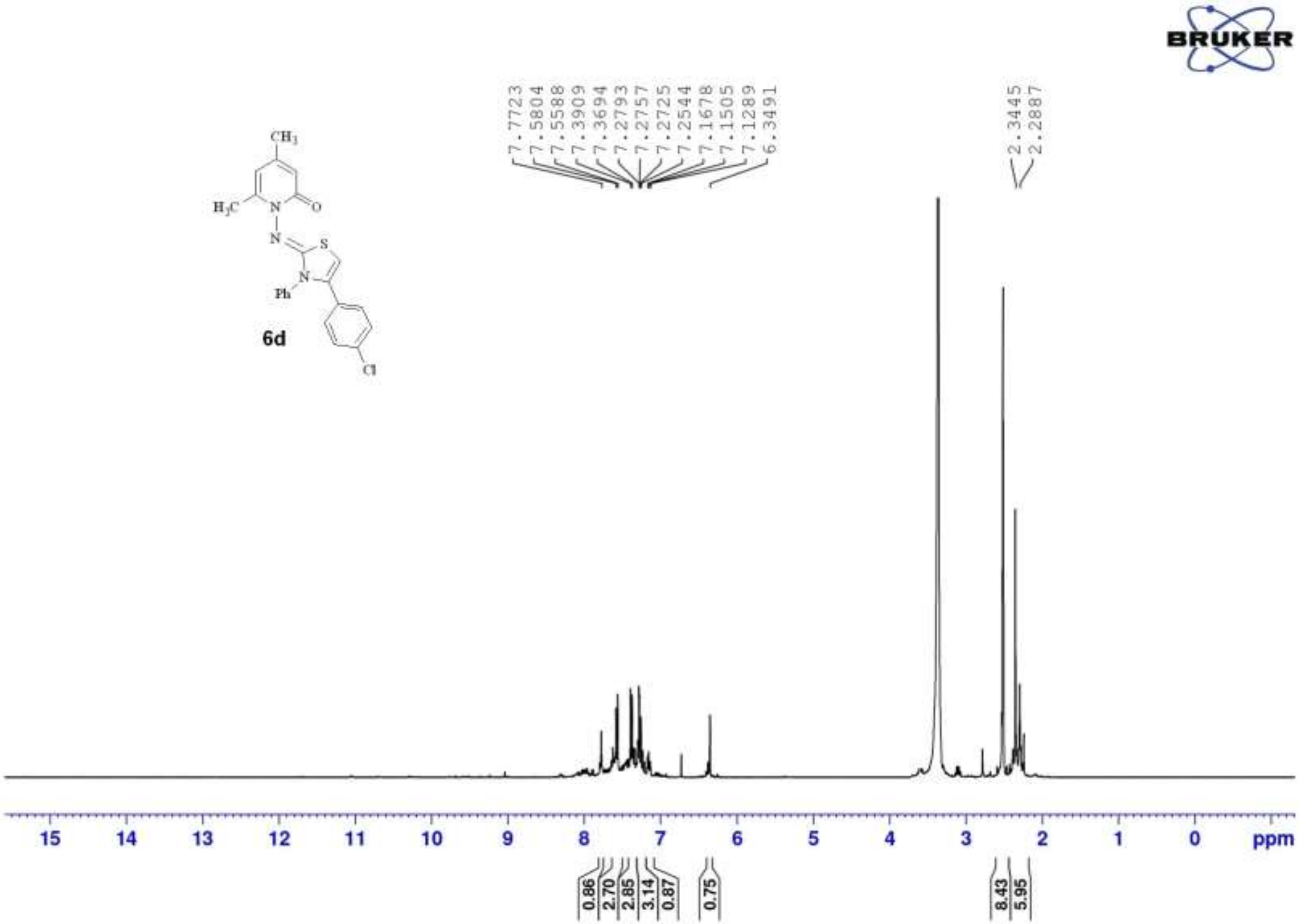

^{1}H-NMR of compound **6d**

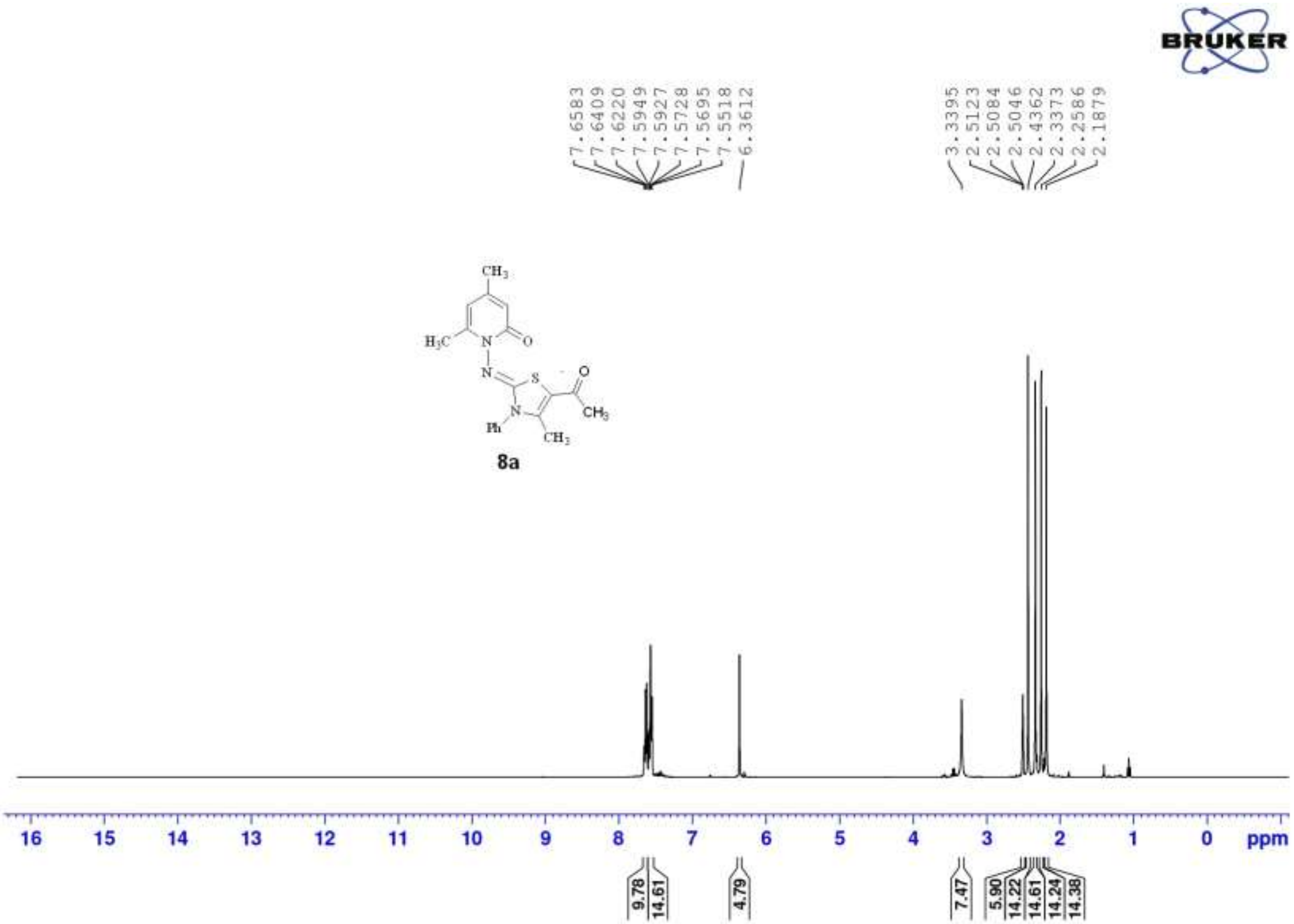

^{1}H-NMR of compound **8a**

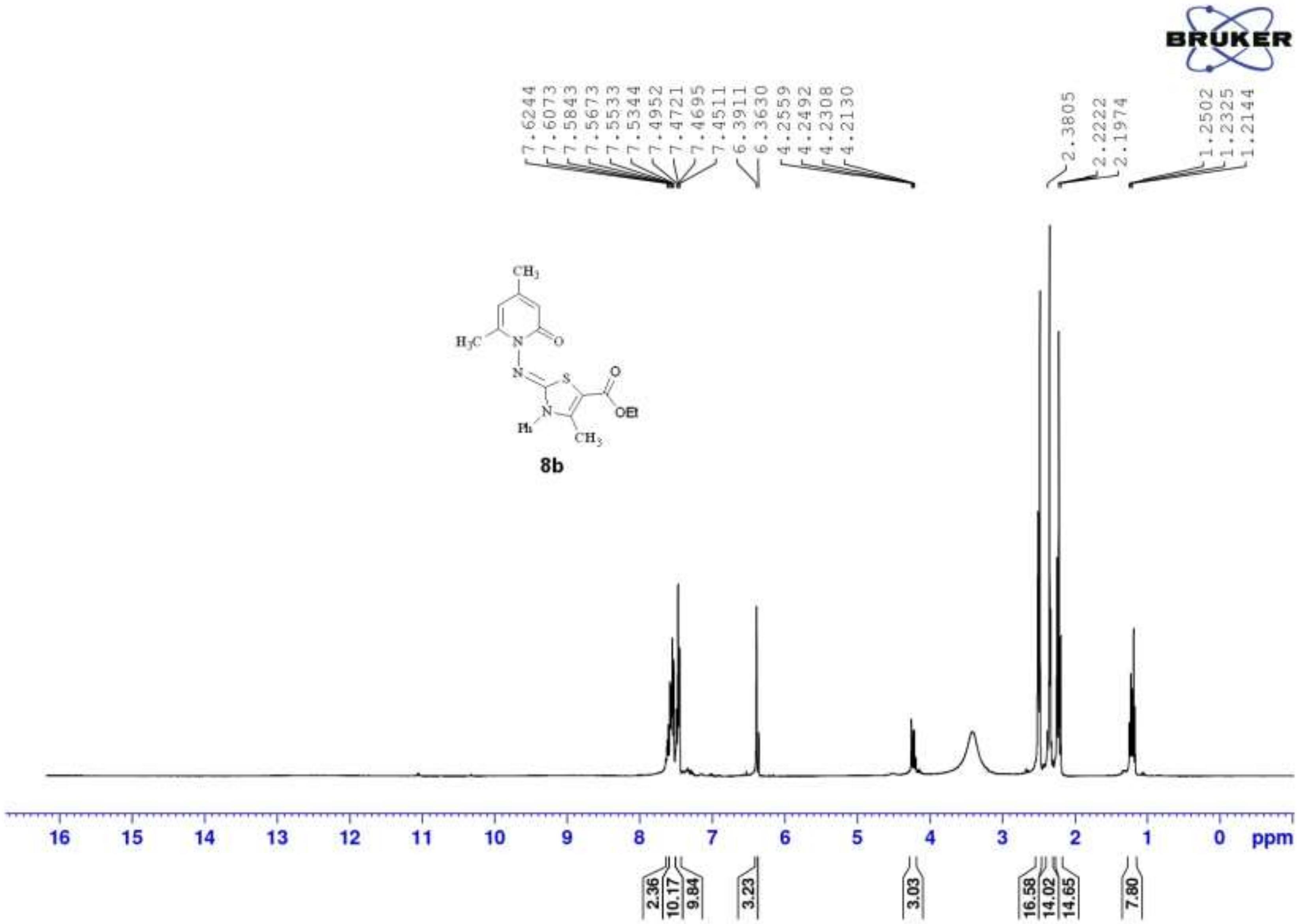

^{1}H-NMR of compound **8b**

Al-Azhar University
The Regional Center for Mycology & Biotechnology

Evaluation of cytotoxicity against HCT-116 cell line

Requester Data:

Name:
Authority: **Faculty of Science, Cairo University**
Sample Code: **4h**

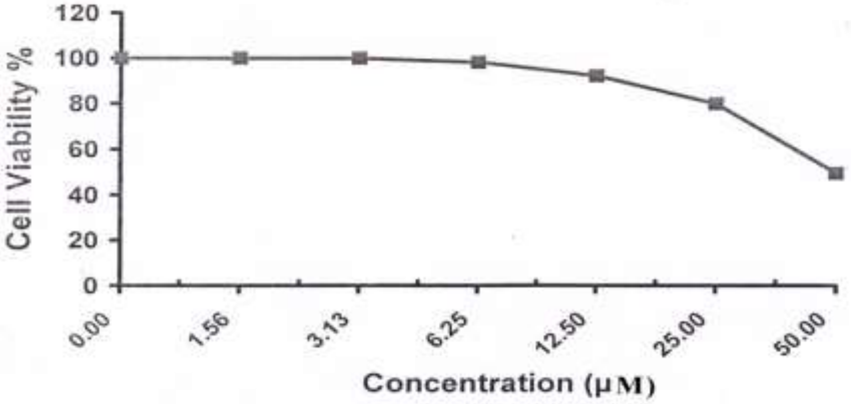

Sample conc.	Viability %
50	49.62
25	80.14
12.5	92.39
6.25	98.26
3.125	100
1.56	100
0	100.00

Comment:

Inhibitory activity against colon carcinoma cells was detected under these expeimental conditions with IC_{50} = 2.03 μM

Investigator (s) **Director**

Al-Azhar University
The Regional Center for Mycology & Biotechnology

Evaluation of cytotoxicity against HepG-2 cell line

Requester Data:

Name:
Authority: **Faculty of Science, Cairo University**
Sample Code: **4h**

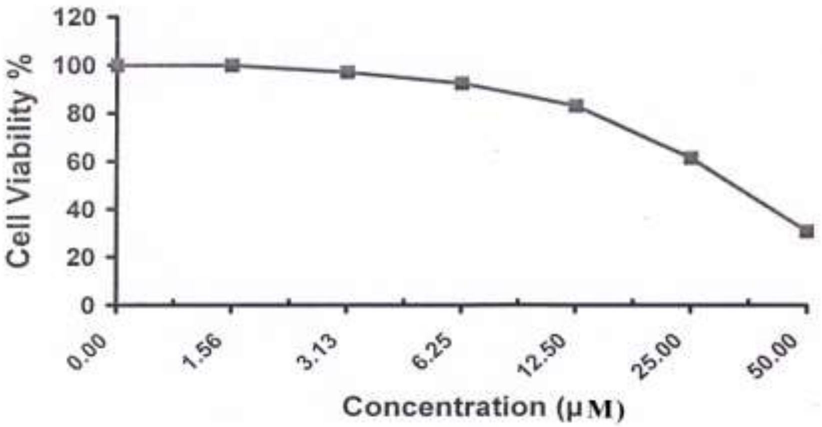

Sample conc.	Viability %
50	30.84
25	61.43
12.5	83.12
6.25	92.44
3.125	97.13
1.56	100.00
0	100

Comment:

Inhibitory activity against hepatocellular carcinoma cells was detected under these expeimental conditions with $IC_{50} = 2.17 \mu M$

Investigator (s)

Director
